# Weight Gain Prevention Outcomes From a Pragmatic Digital Health Intervention With Community Health Center Patients: Randomized Controlled Trial

**DOI:** 10.2196/50330

**Published:** 2024-03-28

**Authors:** Hailey N Miller, John A Gallis, Miriam B Berger, Sandy Askew, Joseph R Egger, Melissa C Kay, Eric Andrew Finkelstein, Mia de Leon, Abigail DeVries, Ashley Brewer, Marni Gwyther Holder, Gary G Bennett

**Affiliations:** 1 School of Nursing Johns Hopkins University Baltimore, MD United States; 2 Department of Biostatistics & Bioinformatics Duke University Durham, NC United States; 3 Duke Global Health Institute Duke University Durham, NC United States; 4 Duke Digital Health Science Center Duke University Durham, NC United States; 5 Department of Pediatrics Duke University Durham, NC United States; 6 Duke-NUS Medical School Singapore Duke Global Health Institute Duke University Durham, NC United States; 7 Caraway New York, NY United States; 8 Medical Home Network Chicago, IL United States; 9 Piedmont Health Services, Inc Chapel Hill, NC United States; 10 Department of Family Medicine University of North Carolina at Chapel Hill Chapel Hill, NC United States; 11 Trinity College of Arts & Sciences Duke University Durham, NC United States

**Keywords:** weight gain prevention, digital health, pragmatic clinical trial, primary care, health disparities, obesity, obese, prevalence, weight management, overweight, intervention

## Abstract

**Background:**

The prevalence of obesity and its associated comorbidities continue to rise in the United States. Populations who are uninsured and from racial and ethnic minority groups continue to be disproportionately affected. These populations also experience fewer clinically meaningful outcomes in most weight loss trials. Weight gain prevention presents a useful strategy for individuals who experience barriers to weight loss. Given the often-limited weight management resources available to patients in primary care settings serving vulnerable patients, evaluating interventions with pragmatic designs may help inform the design of comprehensive obesity care delivered in primary care.

**Objective:**

This study aims to evaluate the effectiveness of Balance, a 2-arm, 12-month pragmatic randomized controlled trial of a digital weight gain prevention intervention, delivered to patients receiving primary care within federally qualified community health centers.

**Methods:**

Balance was a 2-arm, 12-month pragmatic randomized controlled trial of a digital weight gain prevention intervention delivered to individuals who had a BMI of 25-40 kg/m^2^, spoke English or Spanish, and were receiving primary care within a network of federally qualified community health centers in North Carolina. The Balance intervention was designed to encourage behavioral changes that result in a slight energy deficit. Intervention participants received tailored goal setting and tracking, skills training, self-monitoring, and responsive health coaching from registered dietitians. Weight was measured at regular primary care visits and documented in the electronic health record. We compared the percentage of ≤3% weight gain in each arm at 24 months after randomization—our primary outcome—using individual empirical best linear unbiased predictors from the linear mixed-effects model. We used individual empirical best linear unbiased predictors from participants with at least 1 electronic health record weight documented within a 6-month window centered on the 24-month time point.

**Results:**

We randomized 443 participants, of which 223 (50.3%) participants were allocated to the intervention arm. At baseline, participants had a mean BMI of 32.6 kg/m^2^. Most participants were Latino or Hispanic (n=200, 45.1%) or non–Latino or Hispanic White (n=115, 26%). In total, 53% (n=235) of participants had at least 1 visit with weight measured in the primary time window. The intervention group had a higher proportion with ≤3% weight gain at 6 months (risk ratio=1.12, 95% CI 0.94-1.28; risk difference=9.5, 95% CI –4.5 to 16.4 percentage points). This difference attenuated to the null by 24 months (risk ratio=1.00, 95% CI 0.82-1.20; risk difference=0.2, 95% CI –12.1 to 11.0 percentage points).

**Conclusions:**

In adults with overweight or obesity receiving primary care at a community health center, we did not find long-term evidence to support the dissemination of a digital health intervention for weight gain prevention.

**Trial Registration:**

ClinicalTrials.gov NCT03003403; https://clinicaltrials.gov/study/NCT03003403

**International Registered Report Identifier (IRRID):**

RR2-10.1186/s12889-019-6926-7

## Introduction

The prevalence of obesity and its associated comorbidities continue to rise in the United States, and populations who are uninsured and from racial and ethnic minority groups continue to be disproportionately affected [1-8]. These populations also experience fewer clinically meaningful outcomes in most weight loss trials, suggesting a need for alternative intervention approaches to contend with obesity and its associated clinical outcomes [9-11]. Weight gain prevention presents a useful—and guidelines-adherent—strategy for individuals who experience barriers to, or have less success with, weight loss. Compared with weight loss, weight gain prevention can be achieved at lower treatment intensity and is well suited for delivery using electronic health technologies, yielding the potential to reach large, high-risk populations at low costs [12].

We previously demonstrated the efficacy of weight gain prevention interventions in a population of Black female individuals in the community health center (CHC) setting [12,13]. The intervention focused on creating a slight energy deficit sufficient to offset weight gain using medium-intensity digital health strategies. While efficacious, there were concerns about the intervention’s suitability for implementation, as it required significant staff effort and used labor-intensive technologies for participant engagement. Given the often-limited weight management resources available to patients in primary care settings serving vulnerable patients, more frequently evaluating intervention with pragmatic designs may help inform the design of comprehensive obesity care delivered in primary care [14].

We report outcomes from Balance, a 2-arm, 12-month pragmatic randomized controlled trial of a digital weight gain prevention intervention delivered to patients receiving primary care within Piedmont Health Services Inc (Piedmont), a network of Federally Qualified Community Health Centers located in central North Carolina. We hypothesized that, compared with usual care, the intervention arm would have a greater proportion of participants with ≤3% weight gain at 24 months after randomization.

## Methods

### Study Design

Full details of the trial protocol, study population, Balance intervention, and recruitment processes have previously been published [14] and are summarized here. Balance participants were adults with overweight or obesity who received care at participating Piedmont CHCs. Participants were randomized to receive either (1) the 12-month Balance weight gain prevention intervention or (2) a healthy living usual care arm. There were no trial-specific follow-up visits conducted following randomization. Participant outcomes were collected from the Piedmont electronic health record (EHR) at 6, 12, 18, and 24 months after randomization.

### Ethics Approval

Approvals for the trial were obtained by the Duke University institutional review board (2017-0738/D0479) and the Piedmont board in 2016. Verbal informed consent was provided from all participants prior to enrolling in the trial. The trial was registered on ClinicalTrials.gov (NCT03003403) on December 16, 2016. All data presented in this manuscript are deidentified. As Balance was a pragmatic trial with outcomes extracted from the Piedmont EHR and thus did not require participant study visits or follow-up surveys for evaluation, monetary compensation was not provided.

### Study Setting and Sample

Congruent with the trial’s pragmatic design, we sought to limit eligibility criteria to those that were fundamental to intervention implementation or evaluation or represented a significant threat to patient safety. To be included, participants needed to be fluent in English or Spanish and have a BMI between 25 and 40 kg/m^2^ (inclusive) and a weight of 380 pounds or less, as recorded by a provider in the Piedmont EHR within the previous 14 days. The weight eligibility criterion was based on the weight capacity of the connected scales used for intervention delivery. Participants also needed a mobile phone and service plan that could receive weekly trial-related text messages. Exclusion criteria included pregnancy within 12 months, giving birth within 6 months, or breastfeeding within 2 months; having prior weight loss or bariatric surgery; or planning to relocate outside the Piedmont service area during the trial period.

### Trial Recruitment, Screening, and Randomization

Trial recruitment launched in February 2017. In-person recruitment was conducted through a joint effort by Piedmont staff and a Balance research staff member, with Piedmont staff identifying possibly eligible participants and referring them to research staff for questions and additional information. Due to slower-than-expected study recruitment, 4 months into the trial, a new recruitment method was added: an EHR query was used to identify patients who met basic criteria (eg, age and BMI) for Balance and had an upcoming appointment at Piedmont. These patients identified via the EHR query were mailed materials ahead of their appointments and presented trial information during visits to the CHC, if a research team member was present at their appointment time. Interested patients from either recruitment method were prompted to sign an authorization form to allow research staff to assess their medical record for eligibility—and later for evaluation purposes, if enrolled. Following completion of the authorization form, patients underwent additional phone screening to assess criteria not available in their medical records, such as willingness to receive text messages. After eligibility was established, research staff continued to conduct verbal informed consent, enrollment, randomization, and program orientation procedures. Additional details regarding recruitment and screening procedures were previously described [14,15]. Randomization took place concurrently with enrollment between February 2017 and December 2018 using block randomization with stratification by the patient’s CHC within Piedmont Health, with equal allocation to each treatment group.

### Healthy Living Usual Care Arm

Participants randomly assigned to the healthy living usual care arm received enhanced usual care. They received their standard primary care at Piedmont as well as 6 months of automated weekly text messages with healthy living information and printed materials adapted from the National Heart, Lung, and Blood Institute’s *Aim for a Healthy Weight* [16].

### Balance Weight Gain Prevention Arm

The goal of the Balance intervention was to prevent weight gain by encouraging behavioral changes that result in a slight energy deficit. Intervention participants received (1) tailored behavior change goals, (2) skills training materials and videos, (3) weekly self-monitoring prompts for behavioral goal tracking, (4) connected scales for weight self-monitoring, and (5) responsive health and weight coaching from trained registered dietitians.

Behavioral goals were individually assigned and tailored to participants using the interactive obesity treatment approach. The processes for interactive obesity treatment approach goal assignment for Balance have been described elsewhere [14] and have been used in previous primary care obesity treatment trials [13,17,18]. Briefly, participants were administered a simple survey at the start of the trial with a built-in algorithm that creates a net caloric deficit on the backend. For example, participants were asked to rate the frequency of consuming sugary drinks or walking 10,000 steps per day and their perceived confidence to change this behavior. Based on the results of the frequency of each health behavior and their self-efficacy to change it, participants received a set of 3 goals which changed every 8 weeks. They monitored their goal progress by responding to weekly interactive voice response calls and text messages. Participants then received automated, tailored feedback that described their progress, reinforced successes, and offered motivational strategies or short skills training tips.

Throughout the intervention, participants were also asked to weigh themselves daily on a connected scale, as previously described [14,19,20]. Data were transmitted through cellular networks to a database accessible to the research team. A rolling 7-day average of these weights was used to activate the responsive coaching from a Balance dietitian when a participant reached a designated threshold, or zone, of weight gain. If no weights were received across 1 intervention week, study staff would attempt to contact the participant via text or phone to re-engage or troubleshoot.

### Data Collection

Data collection procedures were designed to maximize the use of data collected during routine primary care [21]. Participant sociodemographic characteristics were collected by a brief survey administered as part of the enrollment phone call. All other participant baseline and follow-up data were pulled directly from the EHR (GE Centricity CPS, version 12). Weight measurements in the EHR were recorded in pounds to the nearest 0.1 pound and converted to kilograms for analysis. Height was recorded in inches to the nearest 0.01 inch and converted to centimeters for analysis. BMI was recorded as weight/height (kg/m^2^). EHR data were obtained on all participants between August 6, 2015, and April 30, 2021, including up to 18 months before enrollment through 30.5 months after enrollment.

For participants who became pregnant during the study, all clinical values were censored as of the beginning of their pregnancy. For pregnancies that occurred before enrollment, we censored clinical values from the beginning of pregnancy to 6 months after the end of pregnancy.

### Statistical Methods

Baseline variables were summarized by the intervention arm as means and SDs for continuous variables and as counts and percentages for categorical variables. We used a 2-stage modeling strategy to answer our primary hypothesis. All analyses were intent-to-treat. For the first stage, we estimated the intervention effect on weight using a constrained longitudinal linear mixed-effects model [22], using all available data from between 18 months before enrollment through 30.5 months after enrollment. We explored nonlinearities for the fixed effects of time and random effects of time in the models, guided by the Bayesian information criterion and likelihood ratio tests. The model included fixed effects for time and interactions between time segments and intervention, with a random intercept and random slopes for time. The covariance between random effects was modeled using an unstructured covariance matrix. Randomization was stratified by CHC; thus, we adjusted for CHC in the analyses but did not adjust for other variables. Using this linear mixed-effects model, we estimated mean weight change by intervention arm, and the difference between intervention arms in mean weight change, at 6, 12, 18, and 24 months.

Second, we compared the percentage of ≤3% weight gain—our primary outcome—in each arm at 24 months after randomization using individual empirical best linear unbiased predictors (EBLUPs) from the mixed model [23]. To ensure good predictions, we only used individual EBLUPs from participants with at least 1 EHR weight documented within a 6-month window centered on the 24-month time point (ie, between 21 and 27 months after enrollment). We compared the percentage of ≤3% weight gain in intervention and usual care arms using both a log-binomial model and linear risk model on the EBLUP output, in order to obtain both risk ratios and risk differences, which provide estimates of relative and absolute efficacy, respectively [24]. The extra variability induced by the EBLUPs being predicted from the model was taken into account using a resampling procedure, explained further in the Multimedia Appendices 1 and 2. Sensitivity to the 21- to 27-month window was assessed by obtaining predictions after expanding the window. We additionally used EBLUPs to compare the percentage of ≤3% weight gain in each arm at 6, 12, and 18 months after randomization.

Additional sensitivity analyses included adjusting the model for baseline variables imbalanced across arms, by weight measured in the primary time window (*P*<.10) and removing telehealth visits from the analysis. We also explored effect modification by adding interactions to the primary model with age tertiles; gender; BMI class; race and ethnicity; and preferred language (English or Spanish), each in a separate model. Additionally, in post hoc analyses we added the completion date of the study (before or after COVID-19) as an effect modifier to examine the potential impact of COVID-19 on the intervention effect.

## Results

We randomized 443 participants, of which 223 (50.3%) participants were allocated to the intervention arm and 220 (49.7%) to the usual care arm. Participant demographics are summarized by intervention arm (Table 1). At baseline, participants had a mean BMI of 32.6 (SD 4.0) kg/m^2^ and most participants were Latino or Hispanic (200/443, 45.1%) or non–Latino or Hispanic White (115/443, 26%).

**Table 1:** Balance baseline demographics.

Characteristics			Control (n=220)		Intervention (n=223)		Total (N=443)
Estimated baseline weight (in kg)^a^, mean (SD)			86.8 (14.3)		85.2 (13.1)		86.0 (13.7)
Age (y), mean (SD)			46.8 (13.0)		48.5 (13.6)		47.6 (13.3)
BMI value (kg/m^2^; EHR^b^), mean (SD)			32.9 (3.9)		32.4 (4.0)		32.6 (4.0)
**BMI class (kg/m^2^; EHR), n (%)**							
	25 to <30: Overweight	58 (26.4)		70 (31.4)		128 (28.9)	
	30 to <35: Class I obese	97 (44.1)		87 (39)		184 (41.5)	
	35 to <40: Class II obese	57 (25.9)		64 (28.7)		121 (27.3)	
	40+: Class III obese	8 (3.6)		2 (0.9)		10 (2.3)	
Hours of sleep per 24 hours, mean (SD)			7.1 (1.4)		6.7 (1.3)		6.9 (1.3)
Patient Health Questionnaire-2 score, mean (SD)			0.9 (1.2)		1.0 (1.3)		1.0 (1.3)
**Sex, n (%)**							
	Male	46 (20.9)		43 (19.3)		89 (20.1)	
	Female	173 (78.6)		179 (80.3)		352 (79.5)	
	Male to female transgender	1 (0.5)		1 (0.4)		2 (0.5)	
**Race or ethnicity, n (%)**							
	Hispanic (all races)	98 (44.5)		102 (45.7)		200 (45.1)	
	Non-Hispanic Black	53 (24.1)		53 (23.8)		106 (23.9)	
	Non-Hispanic other or unreported	15 (6.8)		7 (3.1)		22 (5)	
	Non-Hispanic White	54 (24.5)		61 (27.4)		115 (26)	
**Preferred language, n (%)**							
	English	145 (65.9)		141 (63.2)		286 (64.6)	
	Spanish	75 (34.1)		82 (36.8)		157 (35.4)	
**Education, n (%)**							
	Less than high school education	37 (16.8)		45 (20.2)		82 (18.5)	
	High school graduate	65 (29.5)		84 (37.7)		149 (33.6)	
	Some college, vocational degree, or associate’s degree	88 (40)		68 (30.5)		156 (35.2)	
	College graduate or beyond	30 (13.6)		26 (11.7)		56 (12.6)	
**Community health center, n (%)**							
	Carrboro	111 (50.5)		113 (50.7)		224 (50.6)	
	Chapel Hill	28 (12.7)		27 (12.1)		55 (12.4)	
	Moncure	66 (30)		67 (30)		133 (30)	
	Prospect Hill	10 (4.5)		11 (4.9)		21 (4.7)	
	Siler City	5 (2.3)		5 (2.2)		10 (2.3)	
**Recruitment method, n (%)**							
	Via on site efforts only	123 (55.9)		127 (57)		250 (56.4)	
	On site after mailing	43 (19.5)		42 (18.8)		85 (19.2)	
	Via mail or off-site efforts only	54 (24.5)		54 (24.2)		108 (24.4)	
**Leisure-time physical activity, n (%)**							
	Refused	2 (0.9)		0 (0)		2 (0.5)	
	Do not know or not sure	2 (0.9)		1 (0.4)		3 (0.7)	
	No	91 (41.4)		86 (38.6)		177 (40)	
	Yes	125 (56.8)		136 (61)		261 (58.9)	

^a^Estimated from primary linear mixed-effects model.

^b^EHR: electronic health record.

**Figure 1:**
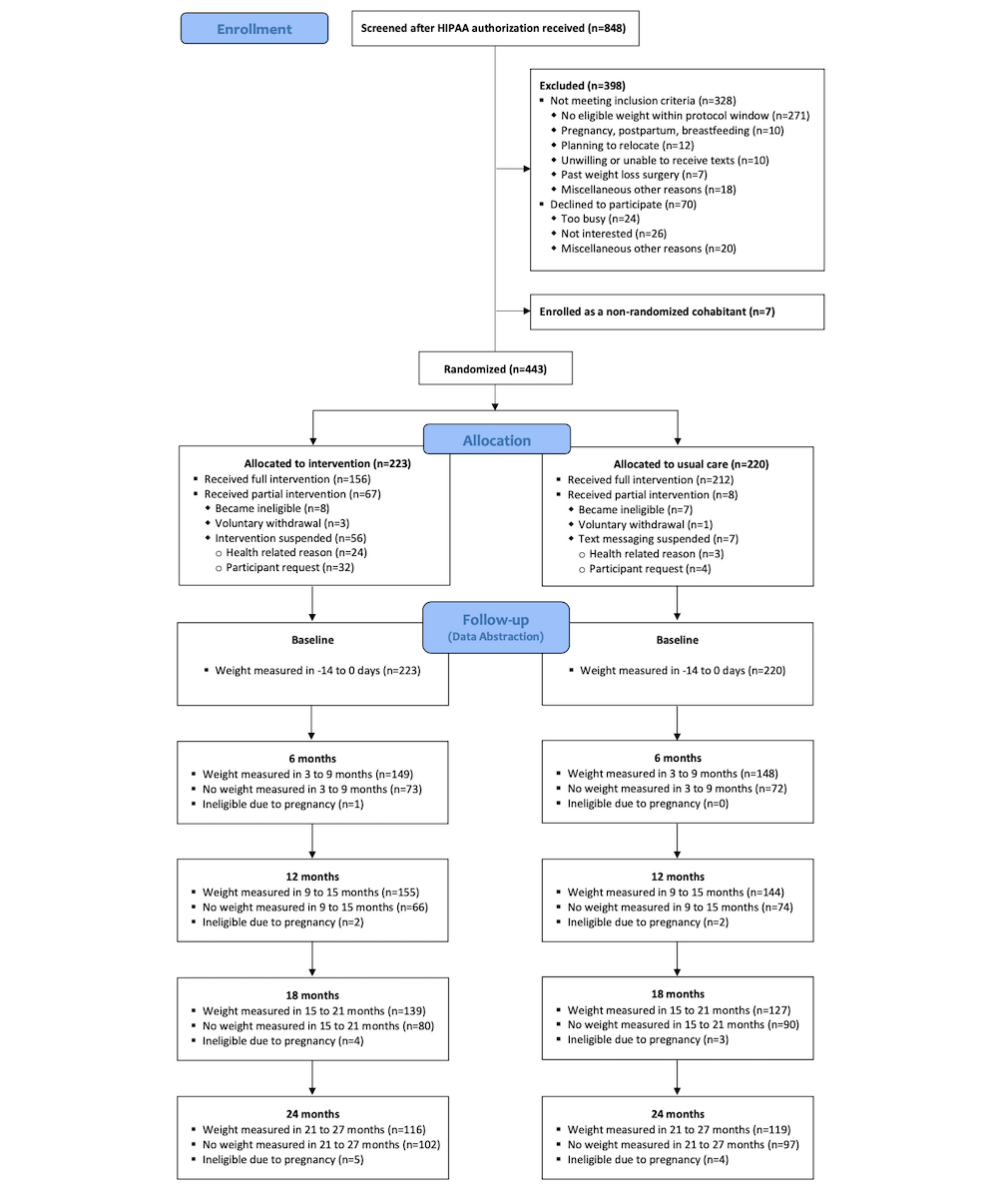
Balance CONSORT (Consolidated Standards of Reporting Trials) flow diagram. For a higher-resolution version of this figure, see Multimedia Appendix 3.

Among our sample, 53% (n=235) of participants had at least 1 visit with weight measured within the primary time window (21 to 27 months; see Figure 1). Participants with visits with weights measured dropped off dramatically during the COVID-19 pandemic (281/443, 63% in April-September 2019 vs 94/443, 21% in April-September 2020; 214/443, 48% in October 2019-February 2020 vs 50/443, 11% in October 2020-February 2021; see Table S1 in Multimedia Appendix 1). This reduced the number of visits in the primary outcome time window since 50% (n=220) of participants had not yet completed all 27 months of follow-up by the start of the pandemic (March 15, 2020). However, there was no significant difference between the arms in the numbers of visits with weights measured in the 21- to 27-month window (119/220, 54% in control vs 116/220, 52% in intervention with a weight measurement in this window; see Table S2 in Multimedia Appendix 1).

**Figure 2:**
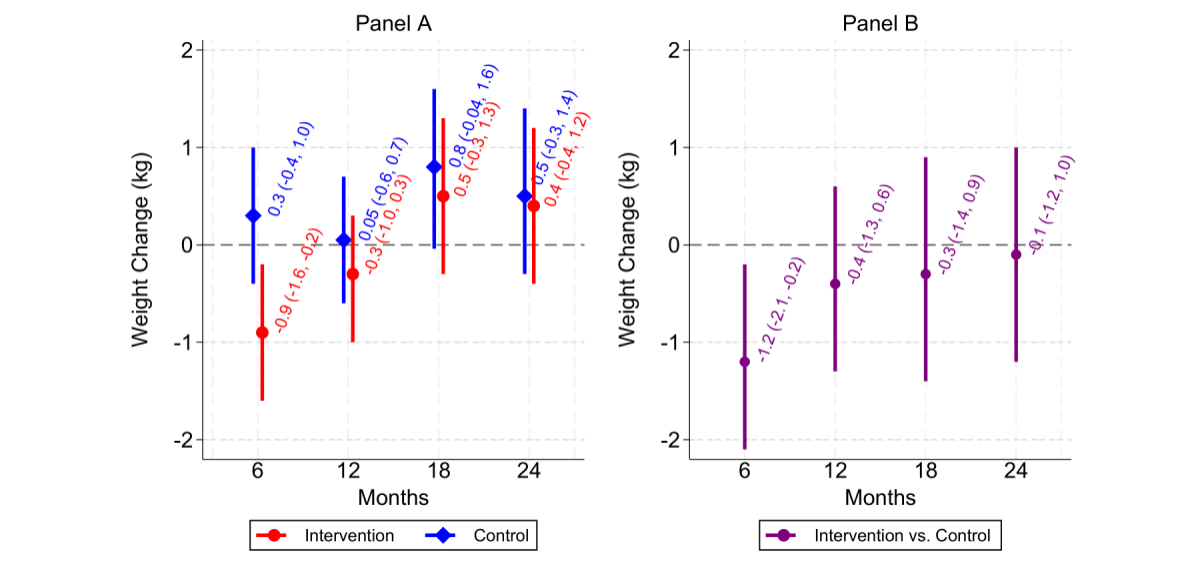
Predicted mean weight change (in kilograms) from baseline in intervention and control separately (panel A), and difference in weight change after baseline comparing intervention to control (panel B). Predictions are from the linear mixed-effects model. In panel B, negative values indicate that the intervention group lost more (or gained less) weight on average than the control group.

**Figure 3:**
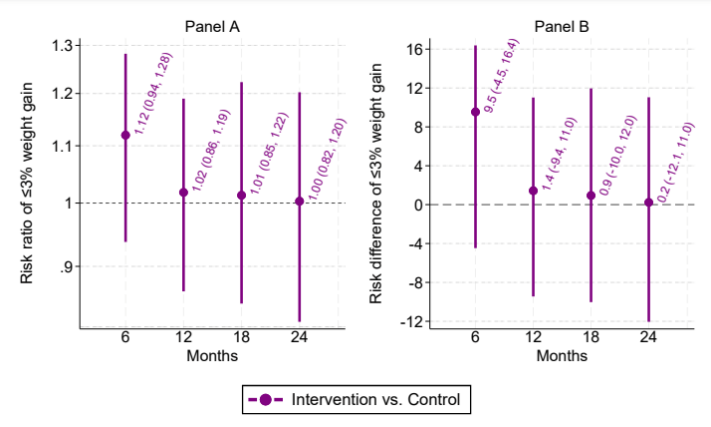
Risk ratio (panel A) and risk difference (panel B) comparing intervention and control on ≤3% weight gain. In panel A, values above 1 indicate that the intervention group had a greater relative probability of ≤3% weight gain versus control. In panel A, values above 0 indicate that the intervention group had a greater absolute probability of ≤3% weight gain versus control.

The linear spline model with knots at 6 months before enrollment; enrollment; and 6, 12, and 18 months after enrollment had the lowest Bayesian information criterion. Compared with the usual care arm, the intervention arm had greater mean weight change at 6 months (–1.2, 95% CI –2.1 to –0.2 kg); however, this estimate was attenuated by 24-months (–0.1, 95% CI –1.2 to 1.0 kg; see Figure 2). The intervention arm had a higher proportion with ≤3% weight gain at 6 months (risk ratio=1.12, 95% CI 0.94-1.28; risk difference=9.5, 95% CI –4.5 to 16.4 percentage points), which also attenuated to the null by 24 months (risk ratio=1.00, 95% CI 0.82-1.20; risk difference=0.2, 95% CI –12.1 to 11.0 percentage points; see Figure 3). Changing the window size around the 24-month time point did not appreciably affect these results (see Figures S1 and S2 in Multimedia Appendix 1). Further descriptive statistics regarding ≤3% weight gain are presented in Table S3 in Multimedia Appendix 1.

Weight measurements were collected at 75 total telehealth visits during the pandemic. After we removed telehealth visits from the model, there were no significant changes to the effect estimates. However, at 18 and 24 months, the effect attenuated toward the null (see Figure S3 in Multimedia Appendix 1).

Table S4 in Multimedia Appendix 1 displays baseline demographics for those with weight measured in the 21- to 27-month window. No variables were imbalanced between those with weight measured in this window versus those without weight measured in this window (*P*<.10); thus, no additional analyses were performed.

Figures S4-S7 in Multimedia Appendix 1 display the results of effect modification analysis. At 6 months, there is evidence of a stronger intervention effect in the 44-57 years age range relative to the ≤43 years age range. No other subgroups across time were significantly different with respect to the intervention effect, including post hoc subgroups based on 24-month completion relative to COVID-19 pandemic onset.

## Discussion

### Principal Findings

We and others have demonstrated the efficacy of weight gain prevention interventions in adults affected by obesity [13,25]. However, in Balance, we did not find long-term evidence to support the dissemination of a digital weight gain prevention intervention to patients receiving primary care at CHCs. Compared with the usual care arm, the intervention arm experienced greater weight loss at 6 months, yet this difference was not clinically meaningful and had attenuated by 24 months. Further, we did not observe a difference between the 2 arms in the proportion of individuals who stayed within 3% of their baseline weight.

### Comparison to Previous Work

Unlike our previous trials using similar approaches resulting in significant weight gain prevention or weight loss [13,18], Balance was designed to use a high level of pragmatism to deliver and evaluate real-world intervention effectiveness. This aligned with the key domains of the Pragmatic-Explanatory Continuum Indicator Summary index, including limited exclusion criteria, nonrestrictive control group, no formal follow-up data collection, and extraction of outcome data directly from the EHR [21]. Pragmatic trial designs vary considerably in the aforementioned domains and these characteristics can assist with contextualizing study findings. For instance, we did not restrict the usual care arm in this trial. As a result, some of these patients may have received medical nutrition therapy or other weight management services from Piedmont registered dietitians or community partners. These efforts may have resulted in the usual care arm experiencing better weight outcomes than expected among a restricted control group, ultimately diminishing trial outcomes.

As described in the methods section and more thoroughly in our protocol paper, the Balance study had a responsive weight and goal coaching protocol. There were 3 zones a participant would be categorized in, depending on their weight change, in which they may have received minimal to no coaching. This differs from our previous weight gain prevention trial that successfully prevented weight gain, in which participants received a standardized coaching protocol that included 20-minute monthly phone calls for 12 months, regardless of weight status [12,13]. It is possible that participants of the Balance intervention would have benefited from this standardized coaching protocol or a higher-intensity intervention.

To our knowledge, no examples exist of other pragmatic weight gain prevention trials in primary care settings among medically vulnerable populations with which to compare our findings. However, there have been several pragmatic weight loss trials implemented in the primary care setting. For example, the PROPEL and REPOWER trials were large, pragmatic randomized weight loss trials implemented in primary care clinics working with medically vulnerable populations and tested a total of 5 weight loss interventions with varying levels of pragmatism [26]. Both trials’ intervention arms observed significant weight loss at 6-months. Yet, not all intervention arms were as successful at producing weight loss, and notably, the arm designed with the highest level of pragmatism observed the least sustained weight loss at 24 months. Along with our study, these findings highlight the challenges of long-term weight management, particularly in pragmatic trials. Future studies might benefit from including additional support for changing motivation, group support, and targeted problem-solving strategies [27,28] to overcome external challenges.

### Limitations and Considerations for Future Research

This study had limitations that should be noted. A potential challenge facing pragmatic weight management trials is the quality of clinic-measured weights. We previously assessed concordance between EHR and research-collected weight data in our earlier weight management interventions and did not find differences in baseline weight, changes in weight from baseline, or differences between intervention and controls in weight change [29]. However, estimates based on EHR weights were generally more variable than weights directly collected by the study team. Another more recent trial demonstrated that weight loss differences observed at 24 months were smaller when using EHR data [30]. The authors offered several explanations for this finding, including the larger variability in follow-up time and the less restrictive procedures for in-clinic weight measurement, such as those observed in Balance. Because we did not collect a secondary measure of weight, we are unable to assess if more controlled weight measurement procedures would have improved our ability to detect differences in weight gain between groups.

We expect that the timeframe of implementation posed challenges to identifying the effects of the Balance intervention on weight outcomes. Namely, the COVID-19 pandemic began during the implementation of the Balance intervention and our 24-month follow-up period. This decreased the number of in-person visits being conducted at the CHCs and, ultimately, impacted follow-up data collection on nearly half of our participants. COVID-19 could have also impacted other weight-related behaviors and risk factors among participants, including physical activity [31,32], food access and dietary quality [33-35], and mental health stressors [36,37]. In addition to COVID-19, there were several other events during the intervention and follow-up timeframe that could have impacted data collection, including 2 major North Carolina hurricanes and immigration reform resulting in increased deportation of undocumented individuals. These factors likely impacted patient appointment attendance and thus the availability of baseline and follow-up EHR data. As such, these events may have affected the precision of the intervention effect estimates for outcomes at 24 months. However, it is unlikely the intervention effect estimates themselves were biased by these events, as there was no difference between the number of visits with weights measured by treatment arms.

Additionally, some components of trial implementation did not align with a pragmatic approach, such as using research staff to conduct recruitment at the health centers. There were also institutional requirements for research compliance that would not be necessary if implemented within a real-world setting. For instance, a Health Insurance Portability and Accountability Act (HIPAA) authorization form was required from each participant before screening could occur, which posed challenges to recruitment ease and timing, as highlighted in our previous article [15]. However, if Balance were disseminated further within Piedmont or other similar CHCs without the inclusion of external research partners, these specific requirements would likely have been lessened or eliminated, thereby decreasing the amount of time required to enroll in the program. Finally, as discussed above, several events, such as COVID-19, occurred during study implementation that impacted data collection. However, as described above, we expect this did not bias effect estimates.

### Strengths

Despite these limitations, there are many notable strengths. The Balance trial was designed with a high level of pragmatism, with limited criteria for eligibility and no study-specific data collection or follow-up procedures, and enrolled a diverse sample of participants. For these reasons, the Balance trial also provides results that can inform decisions in real-world care settings for the management of obesity. Although the intervention did not have a significant impact on weight gain prevention, it is possible that participants adopted healthy dietary and physical activity behaviors that positively impact health and chronic disease management [38]; however, these outcomes were not assessed in this study. Future assessment is warranted given long-term healthy lifestyle changes are possible independent of weight change after participation in a weight gain prevention program [39].

To the best of our knowledge, Balance was also the first weight gain prevention trial to be implemented with a pragmatic design in a primary care setting serving a medically vulnerable population, serving as a strong example of successful partnerships between research teams and CHCs. This sentiment was echoed by Piedmont providers and staff in a qualitative evaluation of Balance [40], in which they stated that early buy-in from providers and staff, respect for the patients and health care setting by the research partnership, and filling a treatment gap for patients all contributed to successful trial implementation. Combined with the trial outcomes, these findings can be used to inform future programs and interventions to be tested and implemented in the CHC setting.

### Conclusions

Balance did not exhibit a positive impact on long-term weight maintenance, yet it provides important information for future pragmatic trials in the primary care setting. Future trials might benefit from including an assessment of the comparator group’s engagement in weight management behaviors and collecting a secondary weight measurement. Researchers may also consider measuring other outcomes associated with chronic disease prevention that are independent of weight, such as changes in diet quality, physical activity, and psychological health.

## Appendix

Multimedia Appendix 1 Estimating variance for the empirical best linear unbiased predictions (EBLUPs).

Multimedia Appendix 2 CONSORT E-HEALTH checklist.

Multimedia Appendix 3 Balance CONSORT (Consolidated Standards of Reporting Trials) flow diagram.

## Abbreviations

CHC: community health center
EBLUP: empirical best linear unbiased predictor
EHR: electronic health record
HIPAA: Health Insurance Portability and Accountability Act
