# Correction: Weight Gain Prevention Outcomes From a Pragmatic Digital Health Intervention With Community Health Center Patients: Randomized Controlled Trial

**DOI:** 10.2196/60137

**Published:** 2024-05-13

**Authors:** Hailey N Miller, John A Gallis, Miriam B Berger, Sandy Askew, Joseph R Egger, Melissa C Kay, Eric Andrew Finkelstein, Mia de Leon, Abigail DeVries, Ashley Brewer, Marni Gwyther Holder, Gary G Bennett

**Affiliations:** 1 School of Nursing Johns Hopkins University Baltimore, MD United States; 2 Department of Biostatistics & Bioinformatics Duke University Durham, NC United States; 3 Duke Global Health Institute Duke University Durham, NC United States; 4 Duke Digital Health Science Center Duke University Durham, NC United States; 5 Department of Pediatrics Duke University Durham, NC United States; 6 Duke-NUS Medical School Singapore Duke Global Health Institute Duke University Durham, NC United States; 7 Caraway New York, NY United States; 8 Medical Home Network Chicago, IL United States; 9 Piedmont Health Services, Inc Chapel Hill, NC United States; 10 Department of Family Medicine University of North Carolina at Chapel Hill Chapel Hill, NC United States; 11 Trinity College of Arts & Sciences Duke University Durham, NC United States

In “Weight Gain Prevention Outcomes From a Pragmatic Digital Health Intervention With Community Health Center Patients: Randomized Controlled Trial” (J Med Internet Res 2024;26:e50330) an error was noted:

In Figure 3, the titles “Panel A” and “Panel B” were missing at the top of the two graphs.

Also in Figure 3, the y-axis title of the second graph:

Risk ratio of ≤3% weight gain

Has been revised to:

Risk difference of ≤3% weight gain

Hence, the revised Figure 3 will be the following:

**Figure 3:**
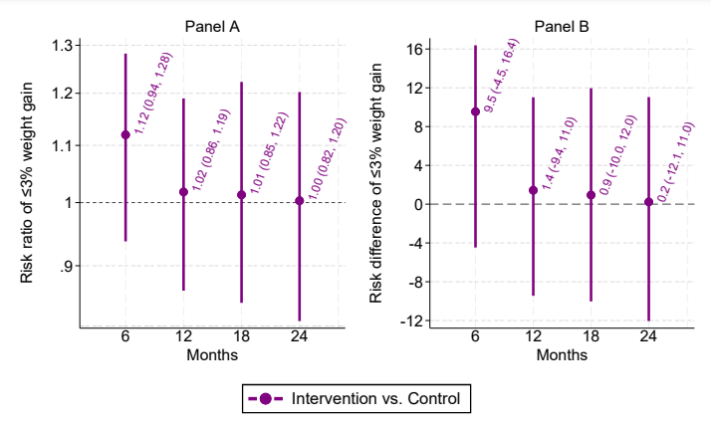
Risk ratio (panel A) and risk difference (panel B) comparing intervention and control on ≤3% weight gain. In panel A, values above 1 indicate that the intervention group had a greater relative probability of ≤3% weight gain versus control. In panel A, values above 0 indicate that the intervention group had a greater absolute probability of ≤3% weight gain versus control.

The correction will appear in the online version of the paper on the JMIR Publications website on May 13, 2024, together with the publication of this correction notice. Because this was made after submission to PubMed, PubMed Central, and other full-text repositories, the corrected article has also been resubmitted to those repositories.

