# Supplementary material for: Weight Gain Prevention Outcomes From a Pragmatic Digital Health Intervention With Community Health Center Patients: Randomized Controlled Trial
Source: J Med Internet Res. 2024 Mar 28;26:e50330. doi: 10.2196/50330 (PMC11009856; doi:10.2196/50330)
Supplement: Multimedia Appendix 1 [file jmir_v26i1e50330_app1.pdf]

Supplementary Material: Estimating variance for the empirical best linear unbiased predictions (EBLUPs)

1. Our EBLUPs method utilizes the predicted weight value (point estimate, under N~, conditional on fixed effect covariates (CHC) and random effect) for each participant at both baseline and 24 months, subtracts these two single values and uses this difference in predicted weight to estimate predicted weight change. This single value is then used in the EBLUPs to dichotomize an individual as above or below the 3% weight change cutoff.
2. This method potentially underestimates the variance in the mean weight change because it utilizes the point estimate of predicted weight at each time point – a value that is, itself, measured with uncertainty.
3. To better incorporate that uncertainty into our EBLUP regression estimates, we did the following:
    a. Record the predicted weight and random effects, along with their standard errors for each participant at both baseline and 24 months, for all participants with a weight measured within the 21 month to 27 month time frame (inclusive).
    b. Draw from a normal distribution around each of these predictions, and compute the EBLUPs using these random draws at both baseline and 24 months.
    c. From these EBLUPs, compute the percentage change and dichotomize at ≤3% weight gain.
    d. Use these EBLUPS to calculate log-risk using log binomial regression and record this log-risk in a new dataset, and repeat this 10,000 times.
    e. Use the distribution of these 10,000 log-risks to determine the 95% confidence interval.
4. We compared this 95% confidence interval to the 95% confidence interval from the statistical model.

Table S1. Visit summary after baseline

	Total (N = 443)	Weight measured (N = 443)	Telehealth visits (N = 443)	Telehealth with weight measured (N = 443)	In-person visit with weight measured (N = 443)
**# with ≥1 visit post-baseline**	424 (95.7%)	423 (95.5%)	76 (17.2%)	45 (10.2%)	423 (95.5%)
**# with ≥1 visit in months 3 to 9**	299 (67.5%)	297 (67.0%)	0 (0.0%)	0 (0.0%)	297 (67.0%)
**# with ≥1 visit in months 9 to 15**	299 (67.5%)	299 (67.5%)	0 (0.0%)	0 (0.0%)	299 (67.5%)
**# with ≥1 visit in months 15 to 21**	274 (61.9%)	266 (60.0%)	13 (2.9%)	7 (1.6%)	263 (59.4%)
**# with ≥1 visit in month 21 to 27**	250 (56.4%)	235 (53.0%)	47 (10.6%)	25 (5.6%)	227 (51.2%)
**# with ≥1 visit pre-covid (Apr-Sep 2019)**	281 (63.4%)	281 (63.4%)	0 (0.0%)	0 (0.0%)	281 (63.4%)
**# with ≥1 visit during covid (Apr-Sep 2020)**	119 (26.9%)	94 (21.2%)	71 (16.0%)	42 (9.5%)	72 (16.3%)
**# with ≥1 visit pre-covid (Oct 2019-Feb 2020)**	217 (49.0%)	214 (48.3%)	0 (0.0%)	0 (0.0%)	214 (48.3%)
**# with ≥1 visit during covid (Oct 2020-Feb 2021)**	51 (11.5%)	50 (11.3%)	12 (2.7%)	10 (2.3%)	42 (9.5%)
**Number of visits total**					
Mean (SD)	7.3 (6.0)	6.9 (5.6)	0.3 (0.7)	0.2 (0.7)	6.7 (5.5)
Min, Max	0.0, 41.0	0.0, 38.0	0.0, 9.0	0.0, 9.0	0.0, 36.0
**Number of visits (months 3 to 9)**					
Mean (SD)	1.6 (1.7)	1.5 (1.7)	0.0 (0.0)	0.0 (0.0)	1.5 (1.7)
Min, Max	0.0, 9.0	0.0, 9.0	0.0, 0.0	0.0, 0.0	0.0, 9.0
**Number of visits (months 9 to 15)**					
Mean (SD)	1.6 (1.8)	1.6 (1.8)	0.0 (0.0)	0.0 (0.0)	1.6 (1.8)
Min, Max	0.0, 12.0	0.0, 12.0	0.0, 0.0	0.0, 0.0	0.0, 12.0
**Number of visits (months 15 to 21)**					
Mean (SD)	1.3 (1.5)	1.2 (1.5)	0.0 (0.2)	0.0 (0.2)	1.2 (1.4)
Min, Max	0.0, 8.0	0.0, 8.0	0.0, 3.0	0.0, 3.0	0.0, 8.0
**Number of visits (months 21 to 27)**					
Mean (SD)	1.3 (1.5)	1.1 (1.4)	0.1 (0.4)	0.1 (0.3)	1.0 (1.4)
Min, Max	0.0, 9.0	0.0, 7.0	0.0, 4.0	0.0, 4.0	0.0, 7.0

Table S2. Summary of visits with weight measured after baseline, by intervention arm

	Control (N = 220)	Intervention (N = 223)
**# with ≥1 visit post-baseline**	206 (93.6%)	217 (97.3%)
**# with ≥1 visit in months 3 to 9**	148 (67.3%)	149 (66.8%)
**# with ≥1 visit in months 9 to 15**	144 (65.5%)	155 (69.5%)
**# with ≥1 visit in months 15 to 21**	127 (57.7%)	139 (62.3%)
**# with ≥1 visit in months 21 to 27**	119 (54.1%)	116 (52.0%)
**# with ≥1 visit pre-covid (Apr-Sep 2019)**	136 (61.8%)	145 (65.0%)
**# with ≥1 visit during covid (Apr-Sep 2020)**	40 (18.2%)	54 (24.2%)
**# with ≥1 visit pre-covid (Oct 2019-Feb 2020)**	106 (48.2%)	108 (48.4%)
**# with ≥1 visit during covid (Oct 2020-Feb 2021)**	23 (10.5%)	27 (12.1%)
**Number of visits total**		
Mean (SD)	6.8 (5.4)	7.0 (5.9)
Min, Max	0.0, 27.0	0.0, 38.0
**Number of visits (months 3 to 9)**		
Mean (SD)	1.5 (1.6)	1.5 (1.7)
Min, Max	0.0, 7.0	0.0, 9.0
**Number of visits (months 9 to 15)**		
Mean (SD)	1.5 (1.7)	1.7 (1.8)
Min, Max	0.0, 9.0	0.0, 12.0
**Number of visits (months 15 to 21)**		
Mean (SD)	1.1 (1.4)	1.3 (1.5)
Min, Max	0.0, 6.0	0.0, 8.0
**Number of visits (months 21 to 27)**		
Mean (SD)	1.1 (1.4)	1.1 (1.5)
Min, Max	0.0, 7.0	0.0, 7.0
**Number of visits (pre-covid [Apr-Sep 2019])**		
Mean (SD)	1.4 (1.5)	1.5 (1.7)
Min, Max	0.0, 8.0	0.0, 12.0
**Number of visits (covid [Apr-Sep 2020])**		
Mean (SD)	0.3 (0.6)	0.5 (1.0)
Min, Max	0.0, 3.0	0.0, 7.0
**Number of visits (pre-covid [Oct 2019-Feb 2020])**		
Mean (SD)	1.0 (1.4)	1.0 (1.4)
Min, Max	0.0, 8.0	0.0, 7.0
**Number of visits (covid [Oct 2020-Feb 2021])**		
Mean (SD)	0.2 (0.5)	0.2 (0.6)
Min, Max	0.0, 4.0	0.0, 4.0

Table S3. Estimated with gained ≤3% baseline weight*

	Control (N = 220)	Intervention (N = 223)	Total (N = 443)
**Gained ≤3% of baseline weight by 6 months**			
Gained more than 3%	30 (20.3%)	16 (10.7%)	46 (15.5%)
Gained less than or equal to 3% of (predicted) baseline weight	118 (79.7%)	133 (89.3%)	251 (84.5%)
Not measured in window	72 (.%)	74 (.%)	146 (.%)
**Gained ≤3% of baseline weight by 12 months**			
Gained more than 3%	29 (20.1%)	29 (18.7%)	58 (19.4%)
Gained less than or equal to 3% of (predicted) baseline weight	115 (79.9%)	126 (81.3%)	241 (80.6%)
Not measured in window	76 (.%)	68 (.%)	144 (.%)
**Gained ≤3% of baseline weight by 18 months**			
Gained more than 3%	35 (27.6%)	37 (26.6%)	72 (27.1%)
Gained less than or equal to 3% of (predicted) baseline weight	92 (72.4%)	102 (73.4%)	194 (72.9%)
Not measured in window	93 (.%)	84 (.%)	177 (.%)
**Gained ≤3% of baseline weight by 24 months**			
Gained more than 3%	29 (24.4%)	28 (24.1%)	57 (24.3%)
Gained less than or equal to 3% of (predicted) baseline weight	90 (75.6%)	88 (75.9%)	178 (75.7%)
Not measured in window	101 (.%)	107 (.%)	208 (.%)

*Based only on participants with weight measured in a 6-month window around the time point of interest.

Table S4. Demographics for those with weight measured, by arm, 21-27 months

	Control (N = 119)	Intervention (N = 116)	p-value
**Age in years**	48.8 (12.8)	50.3 (12.8)	0.375
**BMI value (EHR)**	32.6 (3.9)	32.2 (3.9)	0.486
**BMI class (EHR)**			0.675
25 to < 30: Overweight	36 (30.3%)	39 (33.6%)	
30 to < 35: Class I Obese	50 (42.0%)	44 (37.9%)	
35 to < 40: Class II Obese	30 (25.2%)	32 (27.6%)	
40+: Class III Obese	3 (2.5%)	1 (0.9%)	
**Hours of sleep per 24 hours**	6.9 (1.2)	6.7 (1.4)	0.177
**PHQ-2 Score**	1.0 (1.4)	1.0 (1.4)	0.925
**Gender**	96 (80.7%)	97 (83.6%)	0.555
**Race/ethnicity**			0.774
Hispanic (all races)	58 (48.7%)	52 (44.8%)	
Non-Hispanic white	27 (22.7%)	32 (27.6%)	
Non-Hispanic Black	28 (23.5%)	28 (24.1%)	
Non-Hispanic other/unreported	6 (5.0%)	4 (3.4%)	
**Preferred Language**	45 (37.8%)	41 (35.3%)	0.694
**Education**			0.715
Less than high school education	20 (16.8%)	23 (19.8%)	
High school graduate	40 (33.6%)	44 (37.9%)	
Some college/vocational/associate's	47 (39.5%)	38 (32.8%)	
College graduate or beyond	12 (10.1%)	11 (9.5%)	
**Community Health Center**			0.684
Carrboro	68 (57.1%)	65 (56.0%)	
Chapel Hill	17 (14.3%)	11 (9.5%)	
Moncure	29 (24.4%)	32 (27.6%)	
Prospect Hill	4 (3.4%)	7 (6.0%)	
Siler City	1 (0.8%)	1 (0.9%)	
**Recruitment Method**			0.870
Via on site efforts only	72 (60.5%)	70 (60.3%)	
On site after mailing	23 (19.3%)	20 (17.2%)	
Via mail/off site efforts only	24 (20.2%)	26 (22.4%)	
**Leisure-time physical activity**			0.243
Refused	2 (1.7%)	0 (0.0%)	

	Control (N = 119)	Intervention (N = 116)	p-value
Don't know / Not sure	2 (1.7%)	0 (0.0%)	
No	48 (40.3%)	45 (38.8%)	
Yes	67 (56.3%)	71 (61.2%)	

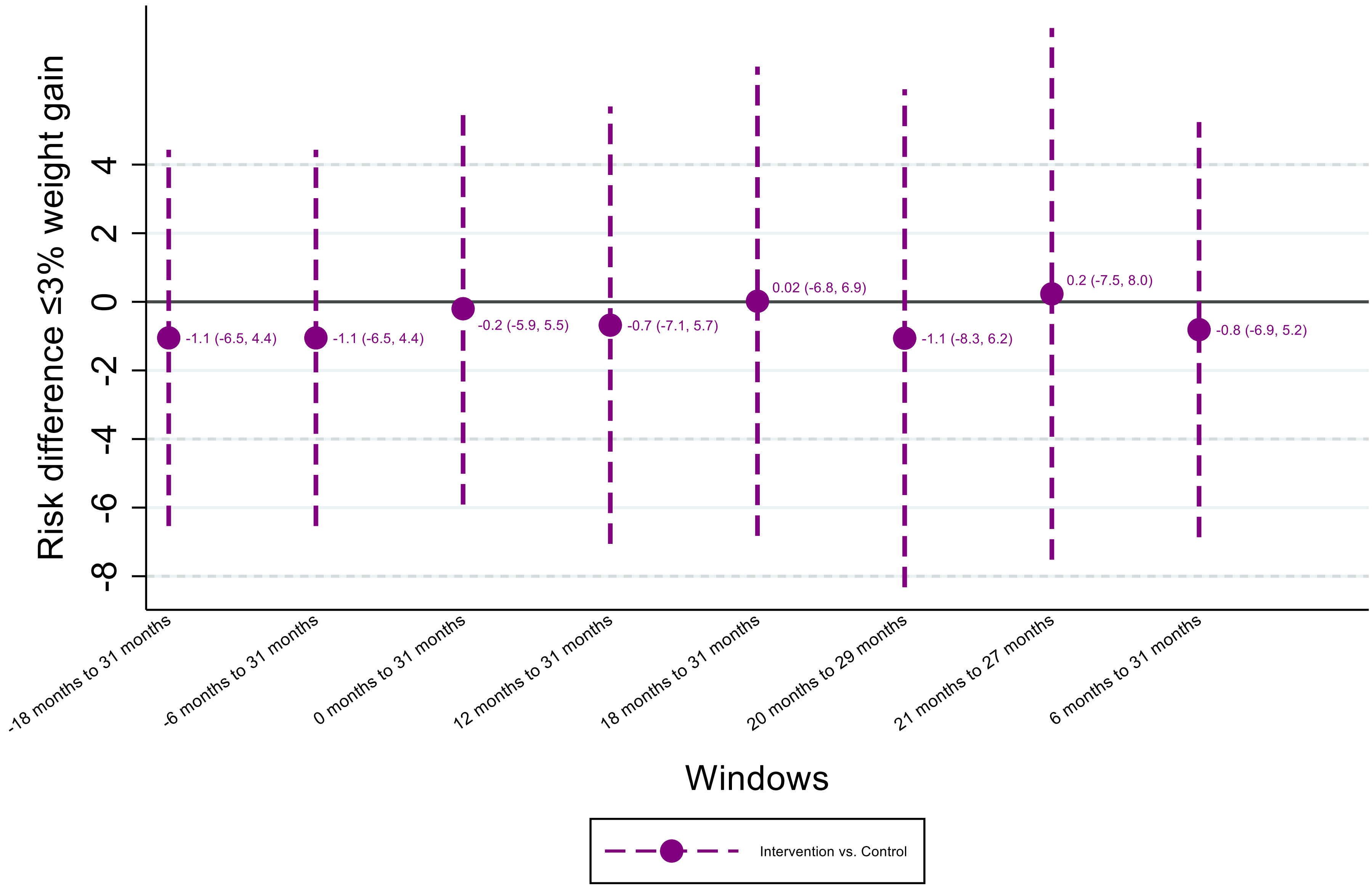

Figure S1. Sensitivity of risk difference to window size. Note that the confidence intervals in these figures are regression-based, not Monte Carlo.

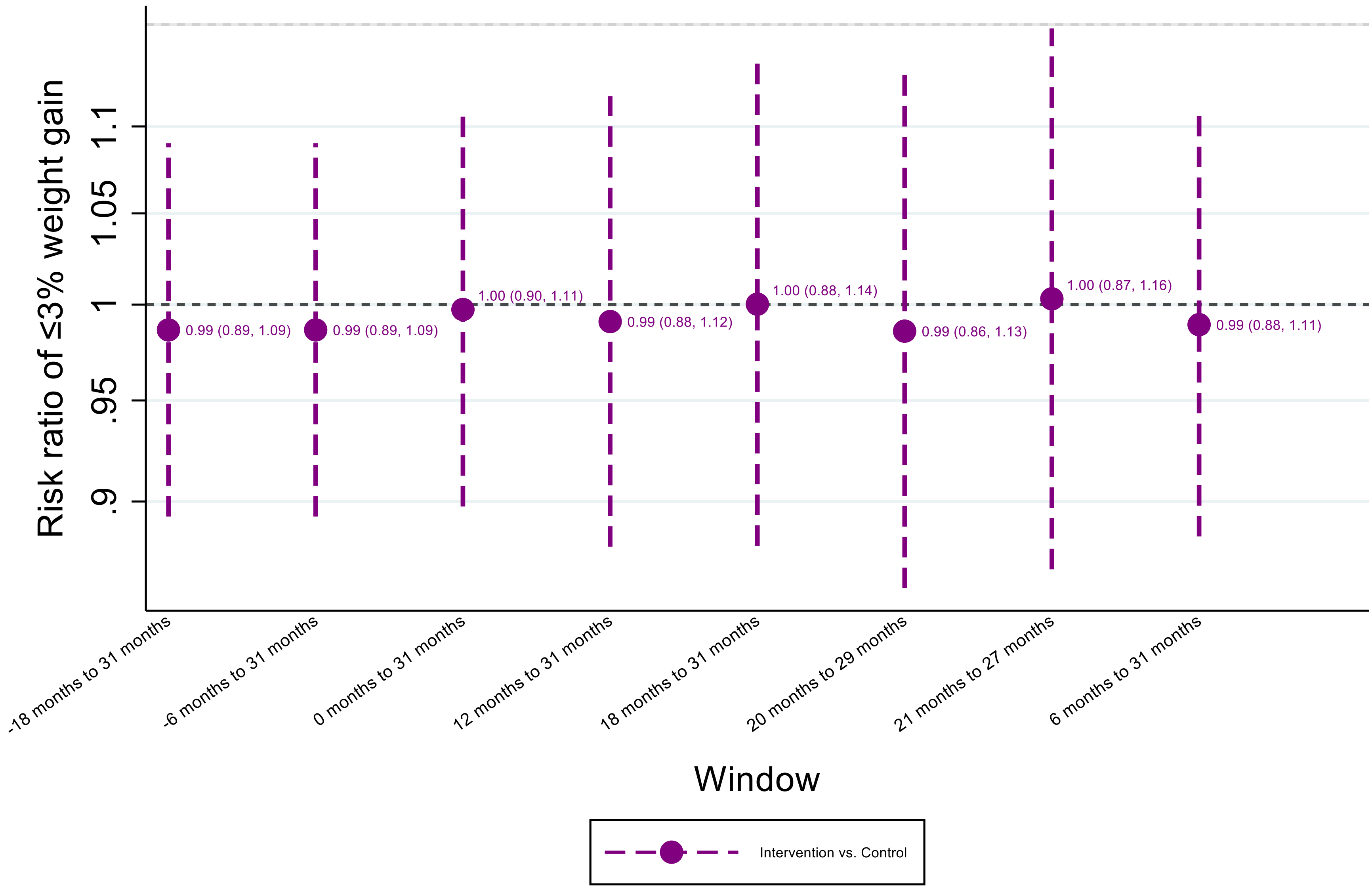

Figure S2. Sensitivity of risk ratio to window size. Note that the confidence intervals in these figures are regression-based, not Monte Carlo.

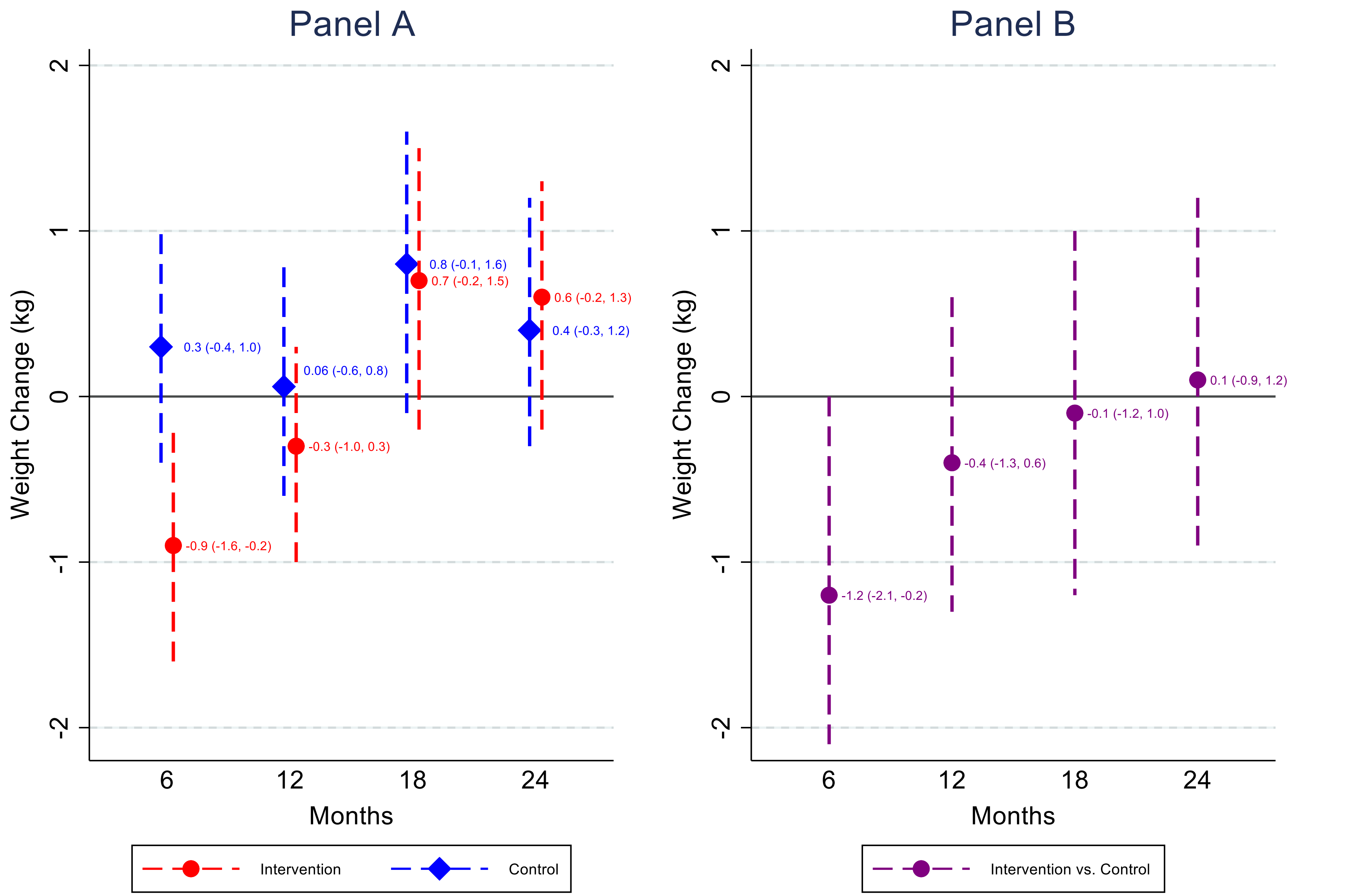

Figure S3. Predicted mean weight change (in kilograms) from baseline in intervention and control separately (panel A), and difference in weight change post-baseline comparing intervention to control (panel B). Predictions are from the linear mixed effects model, with ***all telehealth visits removed*** from the dataset.

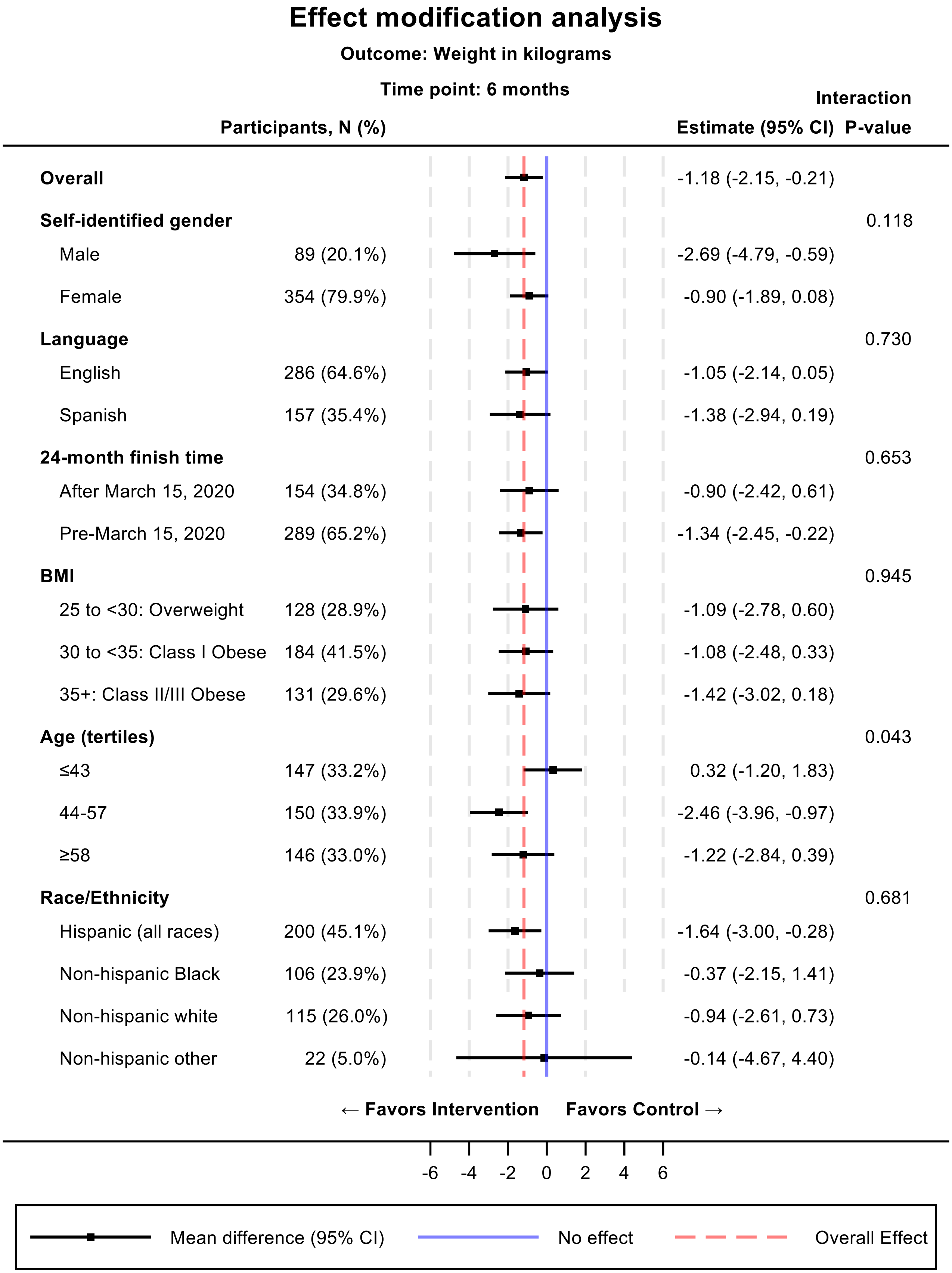

Figure S4. Effect modification analysis at 6 months. Negative values indicate that intervention group lost more (or gained less) weight on average than the control group.

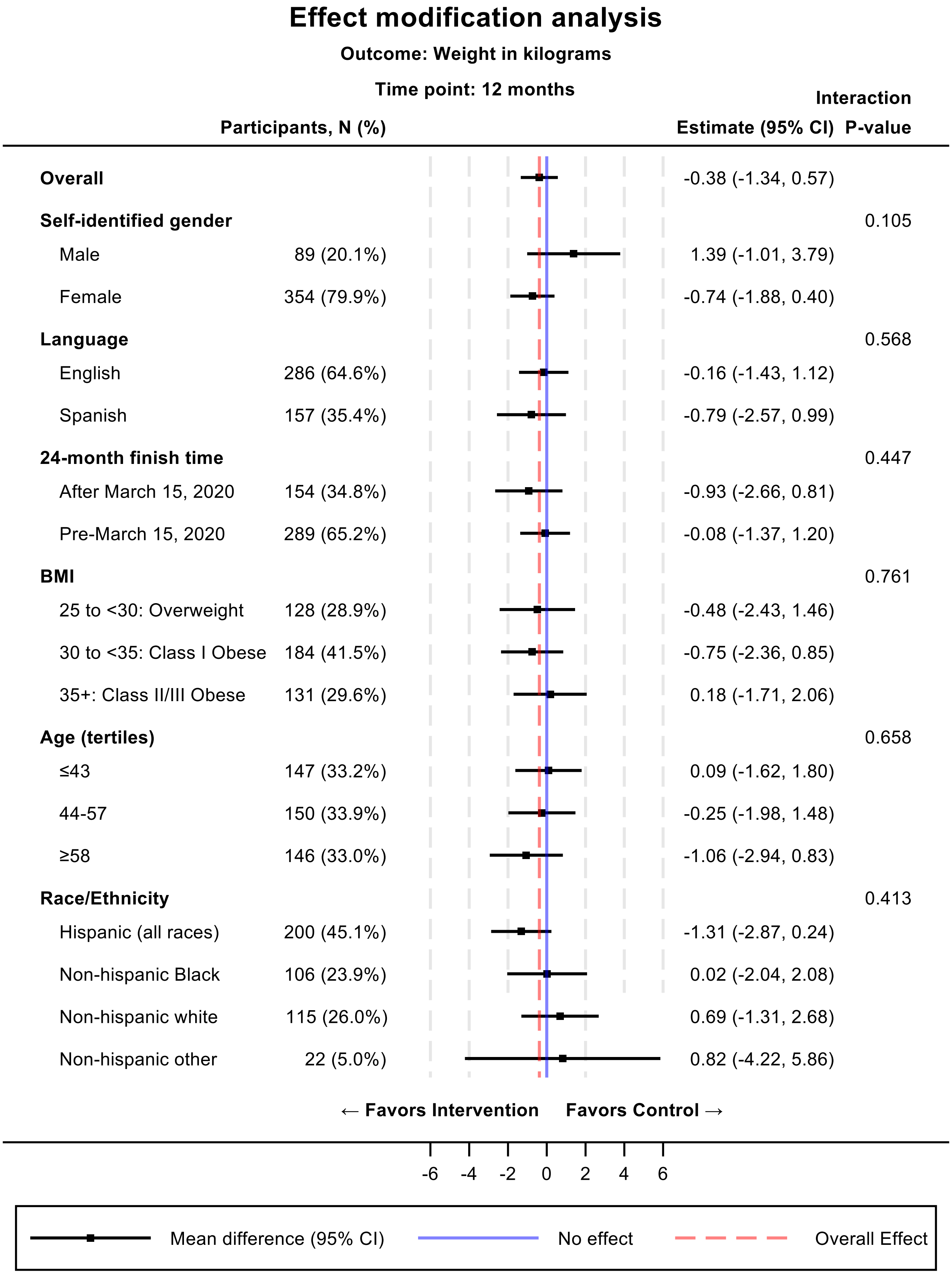

Figure S5. Effect modification analysis at 12 months. Negative values indicate that intervention group lost more (or gained less) weight on average than the control group.

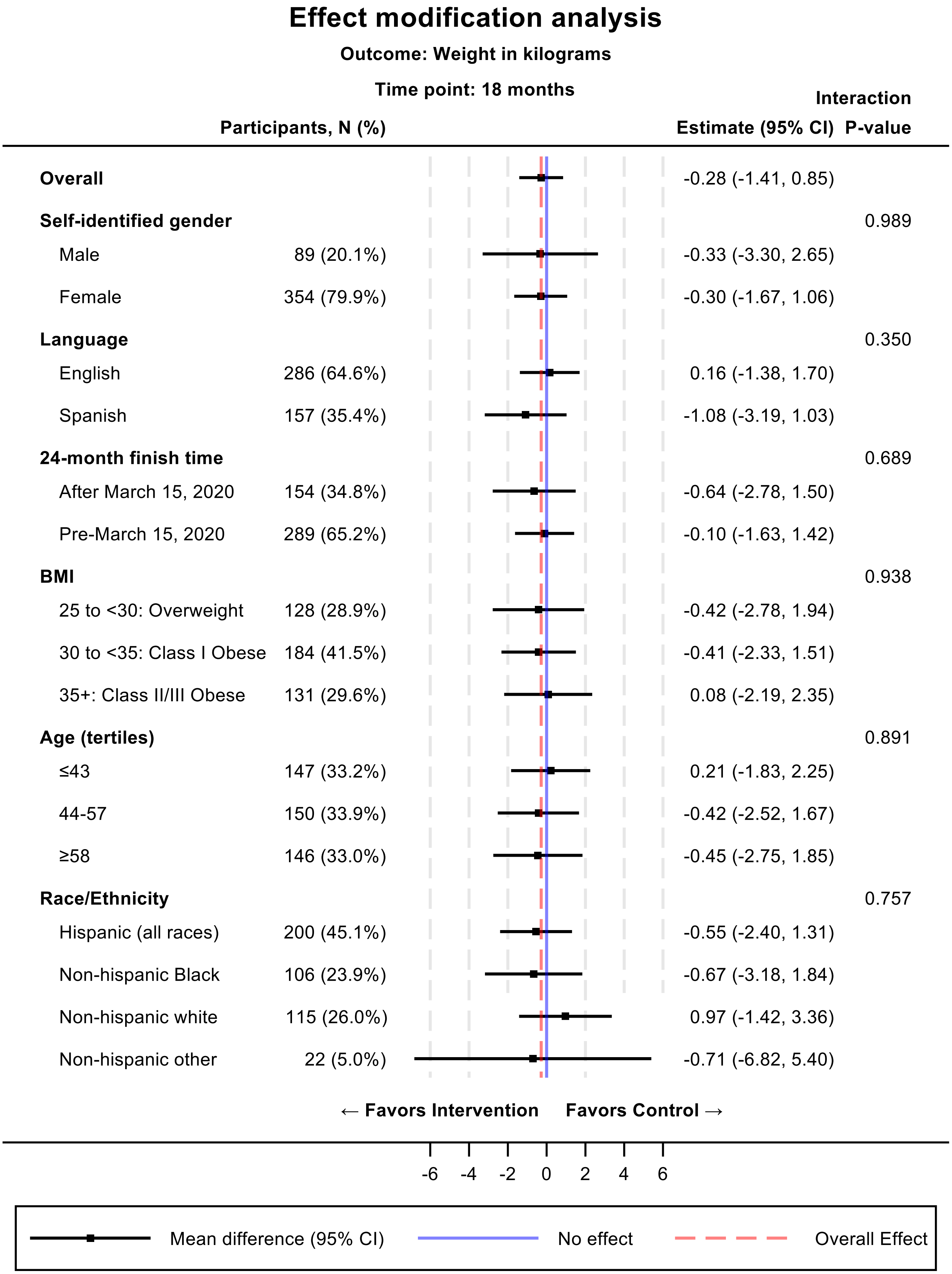

Figure S6. Effect modification analysis at 18 months. Negative values indicate that intervention group lost more (or gained less) weight on average than the control group.

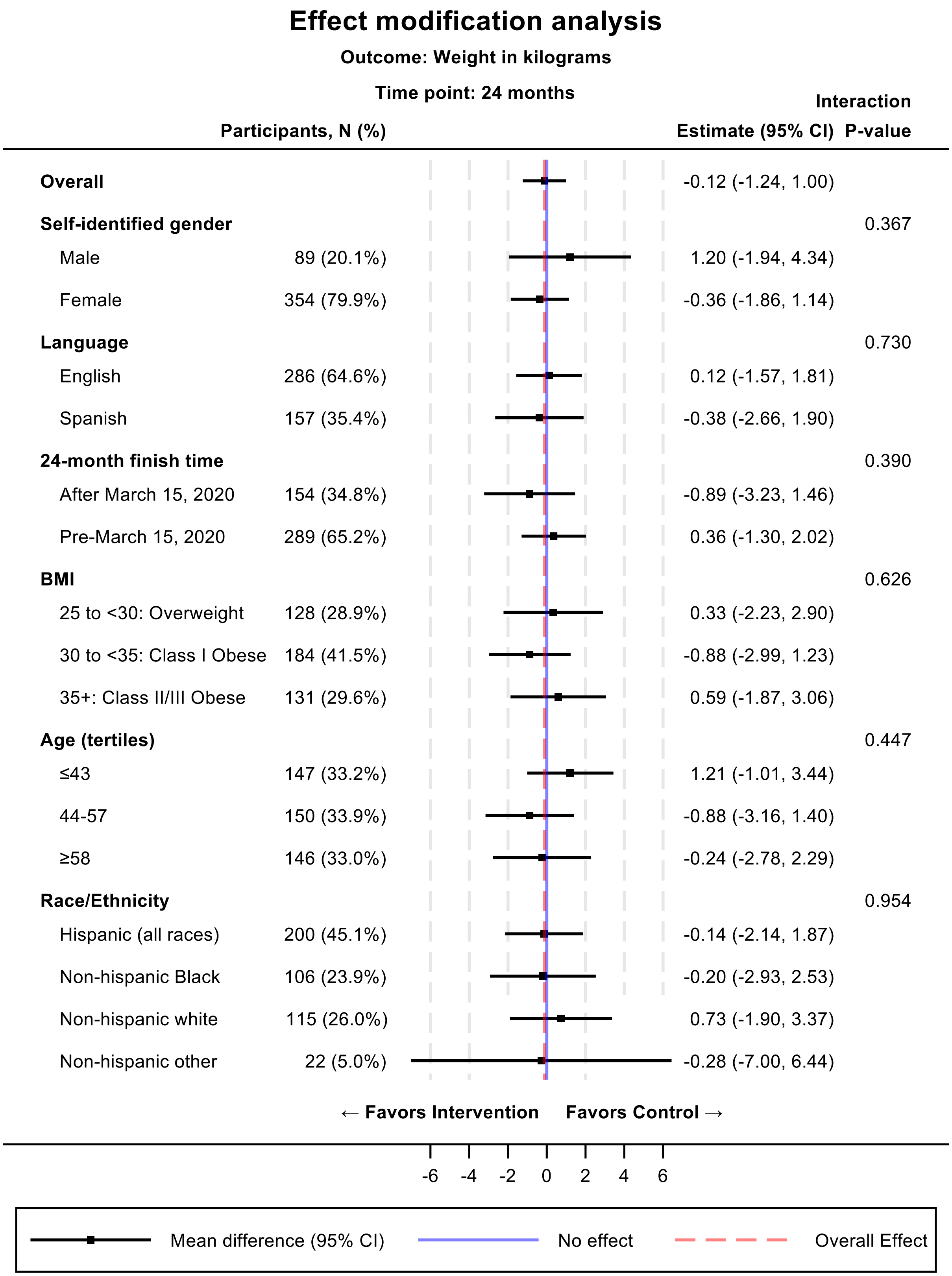

Figure S7. Effect modification analysis at 24 months. Negative values indicate that intervention group lost more (or gained less) weight on average than the control group.
