# Supplementary figures and images for: Weight Gain Prevention Outcomes From a Pragmatic Digital Health Intervention With Community Health Center Patients: Randomized Controlled Trial
Source: J Med Internet Res. 2024 Mar 28;26:e50330. doi: 10.2196/50330 (PMC11009856; doi:10.2196/50330)

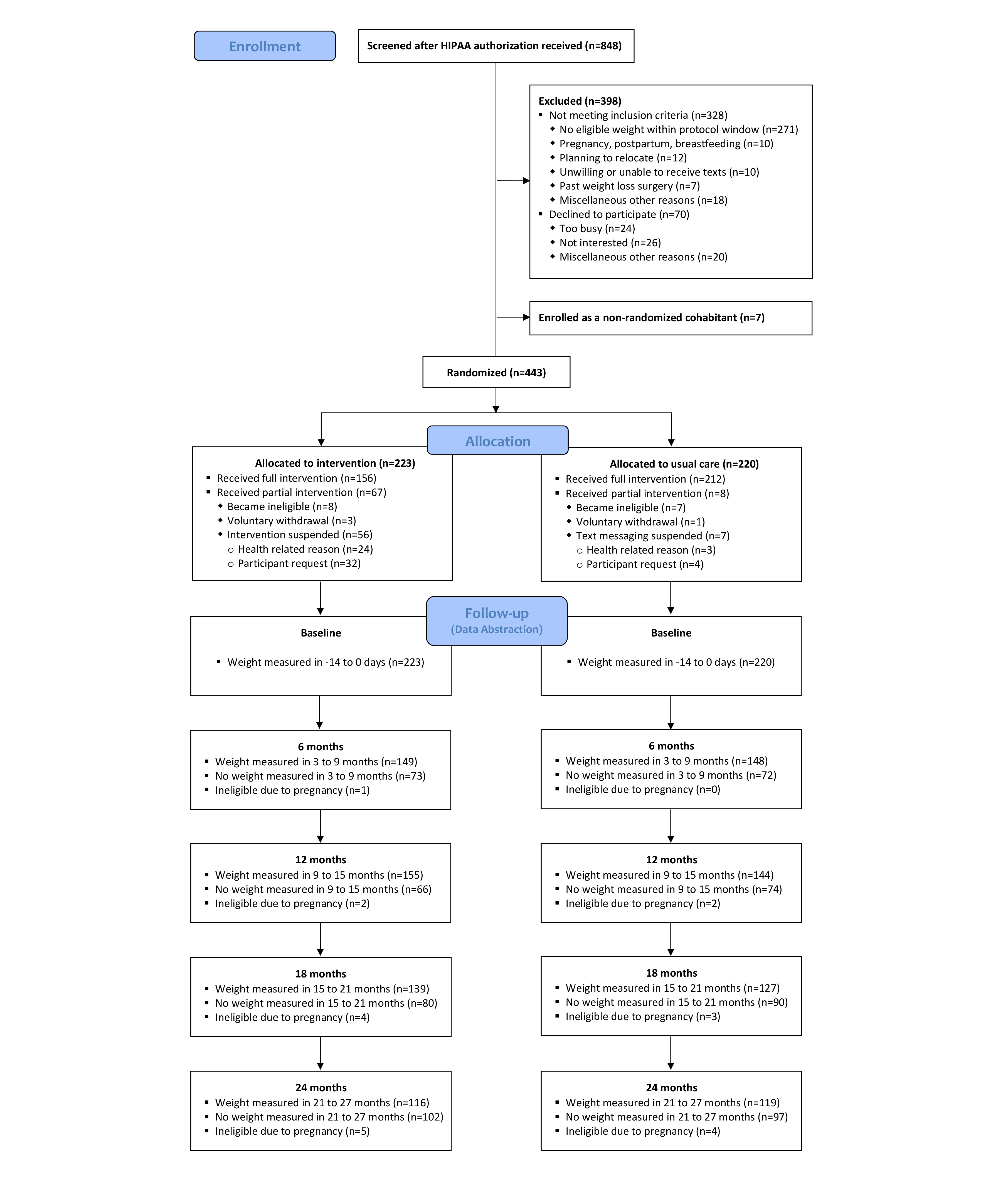

Supplement: Multimedia Appendix 3 [file jmir_v26i1e50330_app3.png]
